# Lignin Valorization: Two Hybrid Biochemical Routes for the Conversion of Polymeric Lignin into Value-added Chemicals

**DOI:** 10.1038/s41598-017-07895-1

**Published:** 2017-08-21

**Authors:** Weihua Wu, Tanmoy Dutta, Arul M. Varman, Aymerick Eudes, Bianca Manalansan, Dominique Loqué, Seema Singh

**Affiliations:** 1 Department of Biomass Science & Conversion Technologies, Sandia National Laboratories, Livermore, CA USA; 2 Joint BioEnergy Institute, Emeryville, CA USA; 3 Department of Bioproducts and Biosystems Engineering, University of Minnesota, St. Paul, MN USA

## Abstract

Naturally, many aerobic organisms degrade lignin-derived aromatics through conserved intermediates including protocatechuate and catechol. Employing this microbial approach offers a potential solution for valorizing lignin into valuable chemicals for a potential lignocellulosic biorefinery and enabling bioeconomy. In this study, two hybrid biochemical routes combining lignin chemical depolymerization, plant metabolic engineering, and synthetic pathway reconstruction were demonstrated for valorizing lignin into value-added products. In the biochemical route 1, alkali lignin was chemically depolymerized into vanillin and syringate as major products, which were further bio-converted into *cis, cis*-muconic acid (ccMA) and pyrogallol, respectively, using engineered *Escherichia coli* strains. In the second biochemical route, the shikimate pathway of Tobacco plant was engineered to accumulate protocatechuate (PCA) as a soluble intermediate compound. The PCA extracted from the engineered Tobacco was further converted into ccMA using the engineered *E. coli* strain. This study reports a direct process for converting lignin into ccMA and pyrogallol as value-added chemicals, and more importantly demonstrates benign methods for valorization of polymeric lignin that is inherently heterogeneous and recalcitrant. Our approach also validates the promising combination of plant engineering with microbial chassis development for the production of value added and speciality chemicals.

## Introduction

Lignin is one of the major components of plant cell wall besides cellulose and hemicellulose, accounting for 10–40 wt% (w/w) of plant cell wall on weight basis^1–3^. Lignin is an amorphous, random branched heteropolymer comprising of phenylpropanoid units^2^. Due to its intrinsic structural heterogeneity and consequent lack of effective routes for lignin valorization, lignin is currently under-utilized and routinely combusted to generate process heat in the paper and pulp industry^4^. However, recent economic studies have suggested that the effective lignin valorization would yield at least 10 times more value as compared to burning it for energy production^5^. As future biorefineries will generate substantial amounts of lignin, the effective valorization of lignin into value-added chemicals, such as vanillin, vanillic acid, catechol, muconic acid, pyrogallol etc., is essential for its economic viability and sustainability.

In the past few years, thermochemical routes for lignin valorization have been under rigorous investigation, mainly focused on producing fuels and aromatics^2, 4, 6–10^. Although the thermochemical routes are of higher efficiency, it is energy intensive, requires expensive catalysts and sometimes toxic chemicals, making the process unsustainable^9–13^. In addition, due to the competing repolymerization pathways, heavier insoluble compounds known as chars and relatively soluble condensed phenols are common byproducts in thermochemical reactions, which greatly hampers the product yield^14^. In nature, some white/brown rot fungi, proteo-, and actinobacteria, can synergistically depolymerize lignin^6, 15^. However, these microbial lignin depolymerization and metabolization require a concerted effort of fungi and bacteria and are extremely slow as compared to thermochemical approaches and suffer from poor carbon economy as huge amount of carbon is lost as carbon dioxide^5, 16^. These factors, especially slow kinetics of lignin depolymerization, make employment of most naturally occurring ligninolytic microbes an ineffective means for lignin depolymerization and highlights the need for microbial consortia for this task. On other hand, lignin valorization through engineered microbes will require the well balanced expression of both 1) lignin depolymerization and 2) metabolization/valorization genes, which is extremely challenging. In addition, expression of these large clusters of genes will impose huge burden on the engineered microbial cells.

**Figure 1:**
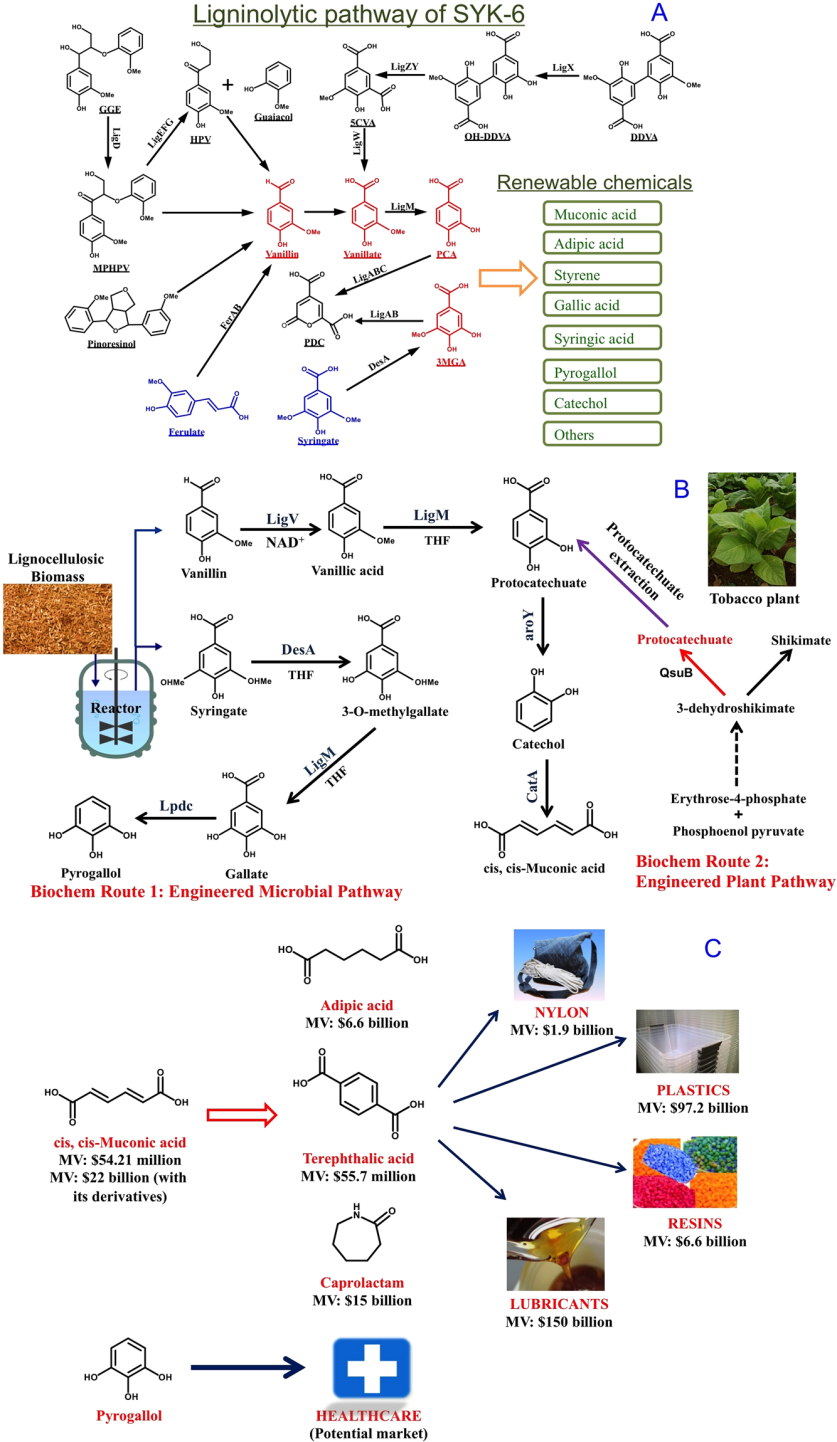
Two hybrid biochemical routes of lignin valorization. (**A**) Ligninolytic pathway of SYK-6. (**B**) The biochemical route 1 integrated the chemical catalysis and biological funneling the lignin into the ccMA and pyrogallol. While the biochemical route 2 combined the lignin biosynthesis engineering in plant Tobacco to accumulate PCA as intermediate compound and the bioconversion of PCA into ccMA through a synthetic chassis. The pathway in the dark green is shared by both routes. (**C**) The market values of some of the products made from muconic acid and pyrogallol.

Aromatic-catabolizing microorganism, such as *Sphingomonas* SYK-6 (thereafter as SYK-6), can metabolize syringate and vanillate, the two main substrates from polymeric lignin breakdown, as a carbon source into smaller central intermediates, such as protocatechuate or catechol, as shown in Fig. 1(A) ^16^. However, utilizing SYK-6 as a host for lignin valorization could be tricky since this will necessitate knock out and over-expression of many genes. Thus, for lignin valorization, a hybrid Chem-Bio route that could benefit from higher efficiency of lignin depolymerization via chemical approaches and higher selectivity of targeted value-added chemicals using microbial approaches is highly desired. The SYK-6 inspired strategy for lignin valorization where an engineered microbial chassis with carefully constructed synthetic pathways further metabolizing the central intermediates and diverting them into designed target chemicals formation could be ground breaking.

Thermochemical processes, such as hydrogen peroxide initiated oxidative lignin depolymerization, is a milder process to valorize lignin^17–19^. Vanillin and other monophenolic compounds, such as syringic acid and vanillic acid, are obtained as major products with yields in the range of 5–10 wt% with respect to the lignin source^17–19^. For example, the vanillin content obtained by the nitrobenzene oxidation of alkali soluble lignin from de-waxed rice straw was 9.82 wt%^19^. The product of lignin oxidation having the highest commercial value is vanillin, corresponding to 5–10% of the total industrial by-products of lignin^18^. To explore the potential of combined strategy, we established a Chem-Synbio hybrid route to convert lignin into value-added products. In this biochemical route, a thermo-chemical pretreatment of Kraft lignin with the hydrogen peroxide was utilized to generate the lignin derived aromatics, vanillin and syringate, two major substrates with cellular degradation pathways in SYK-6. To demonstrate the designed concept, SYK-6 inspired microbial chassis was engineered into *E. coli* to harbor synthetic pathways to convert vanillin into *cis, cis*-muconic acid (ccMA) and syringate into pyrogallol as examples, shown in Fig. 1(B).

In a second approach that avoids the thermochemical approach altogether, making the process environmentally benign without compromising the efficiency and selectivity, we demonstrated a novel hybrid route of lignin valorization combining 1) plant cell wall engineering, and 2) synthetic pathway reconstruction. In this biochemical route, the aromatic amino acid pathway, source of phenylalanine for the lignin biosynthesis pathway in a model Tobacco plant was engineered to convert 3-dehydroshikimate, an intermediate of the shikimate pathway, into protocatechuate (highlighted in red, Fig. 1(B)), which was further extracted from the plant biomass as a substrate for an engineered chassis to convert PCA into value-added chemicals, for example *cis, cis*-muconic acid (ccMA).

The two value added products chosen for the proof of concept demonstration of ‘hybrid chem-bio’ and ‘in-planta-bio’ concepts were muconic acid and pyrogallol. The rationale for this chemical selection were two folds; 1) high value products and, 2) demonstration of conversion routes for both vanillate and syringate. The first product, *cis, cis*-muconic acid, is a precursor for the production of the building block chemicals (adipic acid, terephthalic acid, trimellitic acid) for the synthesis of a various plastics, such as Nylon 6,6, polytrimethylene terephthalate, polyethylene terephthalate, etc^20^., as shown in Fig. 1(C). The traditional process for MA production uses petroleum based feedstocks and high concentrations of heavy metal catalysts, which results in the environmental pollutions. Additionally, the process produces a mixture of *cis,cis*-MA and *cis,trans*-MA from high value catechol and consequently requires the downstream separation and purification with additional cost^21, 22^. It has been reported that the bio-based muconic acid represents a market value of more than $22 billion^23^. The current muconic acid bioproduction technologies are mainly focused on the introduction of heterologous synthetic pathway to drive the intermediate metabolites of the naturally occurring shikimate pathway for the production of muconic acid from glucose^24–27^. Few other studies have been reported that some bacteria can also convert benzoic acid into ccMA via the *ortho*-cleavage of catechol which is one of the intermediates in the beta-ketoadipate pathway^28, 29^, while the benzoic acid source was typically petroleum-based and its production raised many environmental concerns^30^. In this study, we first engineered strain *E. coli* with the introduction of a synthetic pathway, combined “up-pathway” of vanillin degradation (LigV, LigM) in *Sphingomonas* SYK-6 and a protocheuate decarboxylase (aroY) and a catechol dioxygenase (CatA), as shown in Fig. 1(B) and Table 1. For the first time, the engineered strain demonstrated the bioconversion of vanillin into ccMA at high yield.

**Table 1:** Genes used in this study.

Gene	Accession Number	Amino Acids	Function	Source
*aroY*	BAH20873	502	Protocatechuate decarboxylase	*Klebsiella pneumonia subsp*.
*desA*	BAK67175	462	Syringate O-demethylase	*Sphingobium sp. SYK-6*
*Lpdc*	Lp_2945	491	Gallate decarboxylase	*Lactobacillus plantarum WCFS1*
*ligV*	BAK65381.1	480	Vanillin dehydrogenase	*Sphingobium sp. SYK-6*
*ligM*	BAK65949.1	471	3-O-methylgallate-O-demethylase	*Sphingobium sp. SYK-6*
*CatA-pmt2*	BAA07037	311	Catechol 1,2-dioxygenase	*Pseudomonas putida mt-2*
*CatA-ac*	GAM30370	306	Catechol 1,2-dioxygenase	*Acinetobacter calcoaceticus*
*QsuB*	YP_001137362.1	618	3-dehydroshikimate dehydratase	*Corynebacterium glutamicum*

The second product, pyrogallol, is often used in the chemical synthesis to produce biologically active molecules, such as the antibiotics trimethoprim, the muscle relaxant gallamine triethiodide, and insecticide bendiocarb^31^. In some recent studies, pyrogallol was reported to have anti-proliferative effects on some human cancer cell lines^32, 33^, indicating the potential pharmaceutical applications of pyrogallol. Industrial production of pyrogallol involves heating gallic acid in copper autoclaves to trigger the thermal decarboxylation of gallic acid. However, the availability of gallic acid is restricted by its isolation from gall nuts or tara powder derived from the ground seed pod of a tree in Peru^31^. Furthermore, there are no studies on the bioconversion of lignin derived aromatics into pyrogallol. Herein, for the first time, we have introduced the heterologous biosynthesis pathway into *E. coli* for the production of pyrogallol from lignin-derived aromatics.

## Results

### Vanillin and syringic acid are two major products in the lignin catalytic valorization

**Figure 2:**
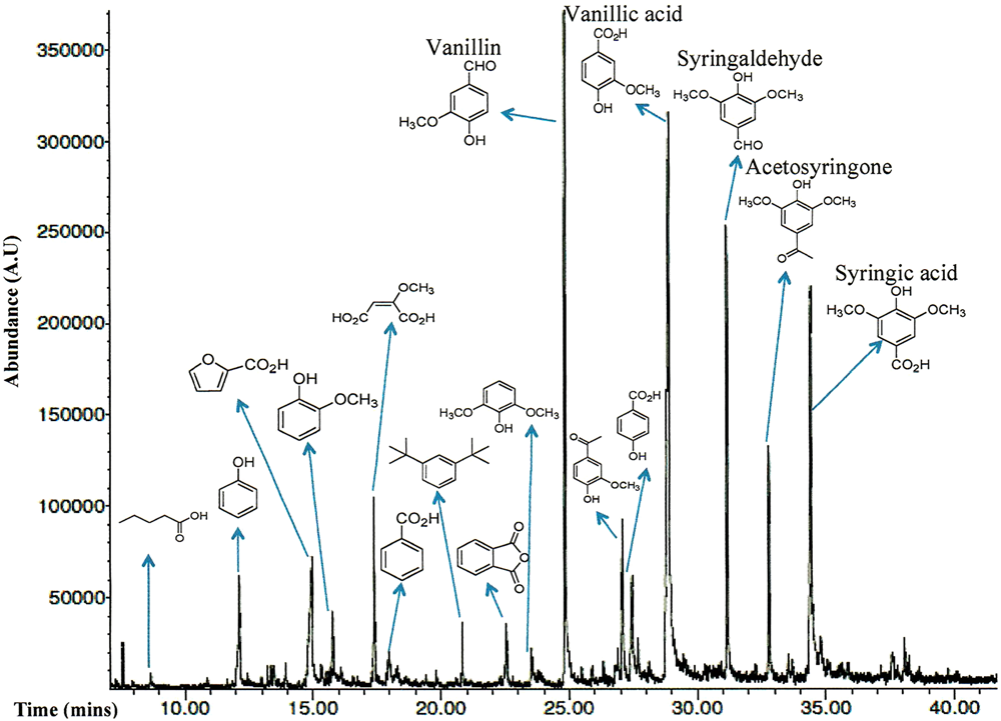
GC spectrum of the components of refined lignin treated by hydrogen peroxide oxidation.

Lignin depolymerization with alkaline hydrogen peroxide is an effective lignin pretreatment method. The depolymerization of lignin is facilitated by the oxidative action of the hydrogen peroxide by fragmenting the lignin macrostructure into a number of low molecular weight compounds. It has been proposed that the hydroperoxy anion generates in the alkaline hydrogen peroxide solution, which reacts with the lignin to produce carboxylic, aldehyde, and phenolic end groups^34, 35^. The depolymerized low molecular weight (mostly monomers) products were analyzed by GC-MS spectrometry after extracting them using ethyl acetate as a solvent. As shown in Fig. 2, it can be observed that the identified depolymerized monomeric products contain mainly carboxylic, phenolic and aldehyde or ketone functional groups. From the GC-MS spectrum, it is also evident that in the depolymerization mixture vanillin, vanillic acid, syringaldehyde, acetosyringone, and syringic acid are the major components. The yields of vanillin/vanillic acid and syringic acid varied within the batches but were in the range of 3–5 wt% and 1–2 wt% from polymeric lignin, respectively. The percentages of other two main components were not quantified due to the lack of suitable standards.

### Bioconversion of vanillin into *cis, cis*-muconic acid

**Figure 3:**
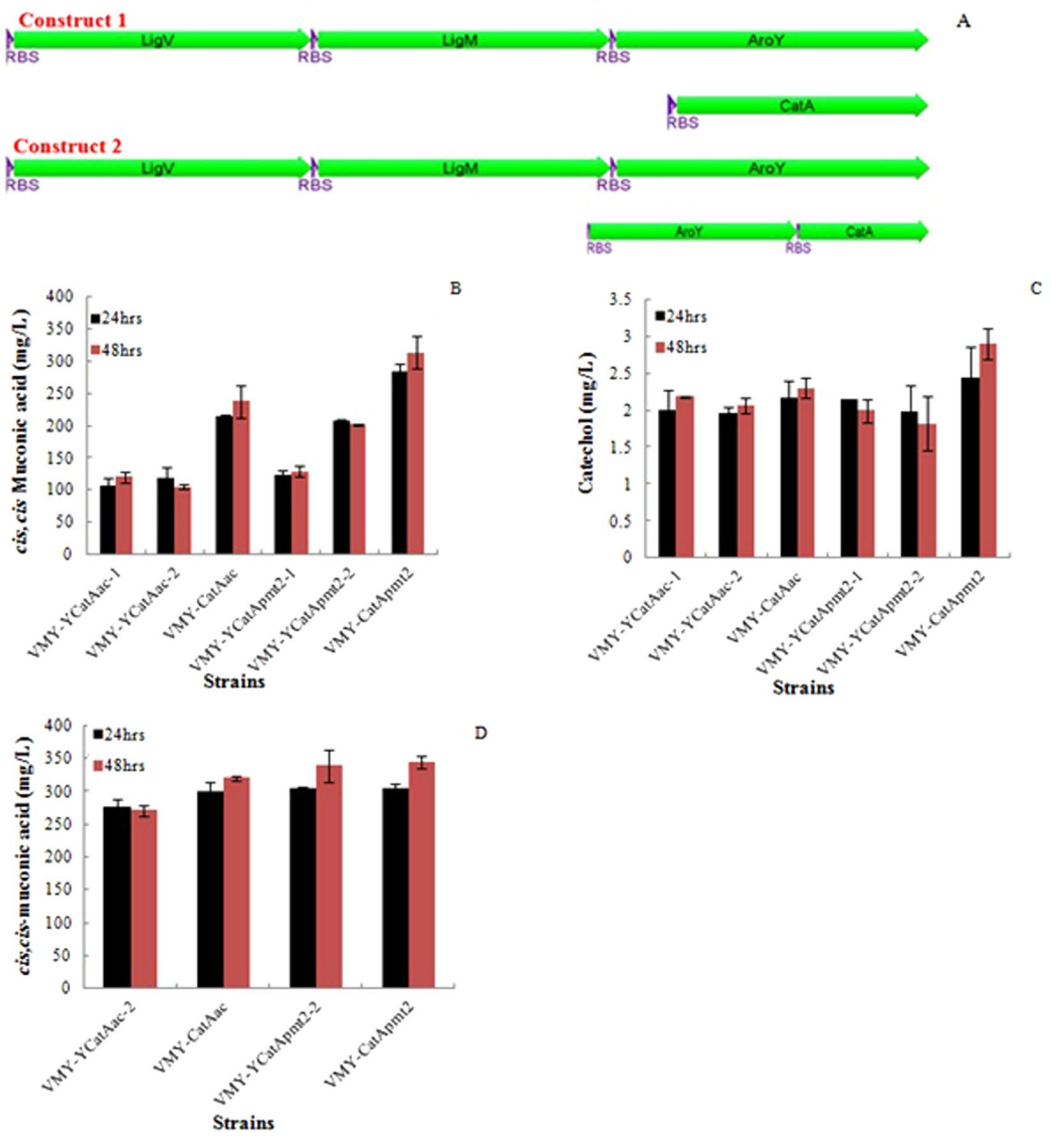
Bioconversion of vanillin into *cis,cis*-Muconic acid. (**A**) synthetic constructs for the bioconversion of vanillin into ccMA. Both constructs harbor four pathway genes:*LigV*, *LigM*, *AroY*, *CatA(CatAac or CatApmt2)*, while construct 2 expressed two copies of *AroY*. Strains VMY-CatA contains construct 1 with *CatA* genes from different sources, represented as VMY-CatAac or VMY-CatApmt2; Two strains containing construct 2 were cultured, represented as VMY-YCatAac 1,2 or VMY-YCatpmt2-1,2, respectively; (**B**) ccMA concentrations from the fermentation broth of strains containg two different constructs; (**C**) Catechol concentrations from the fermentation broth of strains containg two different constructs; (**D**) ccMA concentrations of strains containg two different constructs in whole cell bioconversion.

The current microbial production of *cis,cis*-muconic acid is mainly focused on the manipulation of the aromatic amino acid biosynthesis pathway through converting the intermediate 3-dehydroshikimate via PCA, catechol into *cis,cis*-muconic acid from sugars^25–27, 36–38^. In this study, we developed a novel pathway for *cis, cis*-muconic acid production through bioconversion of the vanillin obtained from hydrogen peroxide catalyzed Kraft lignin. To achieve this goal, four genes (*ligV, ligM, aroY, CatA*) were expressed in the *E. coli* strain DH1 via two different constructs, as shown in Fig. 3(A). Two copies of *CatA* genes (*CatAac* and *CatApmt2*, Table 1) from different bacterial sources were selected and expressed in each construct, respectively. In the construct 1, the three genes *ligV, ligM*, and *aroY* were stacked in a single operon and heterologously expressed in a single plasmid pBbE1a under the control of pTrc promoter while the genes *CatA* were expressed in a plasmid pBbE7K under T7 promoter, respectively. In the construct 2, the first three genes of the operon were expressed in the plasmid pBbE1a under pTrc promoter while a second copy of *aroY* and *CatA* (*CatAac* or *CatApmt2*) were co-expressed in the plasmid pBbE7k under T7 promoter to achieve over-expression of the PCA decarboxylase (aroY) since it is known to be the rate-limiting enzyme step of the ccMA pathway^25, 26^. The results showed that the strains containing either of constructs yielded various amount of *cis, cis*-muconic acid into fermentation broth. The highest titer of *cis,cis*-muconic acid achieved was 314 mg/L by the strain expressing construct 1 containing the gene encoding catechol 1,2-dioxygenase (*CatApmt2*) from the gram negative bacterium *Pseudomonas putida* mt-2^39^, followed by the strain expressing construct 1 (238 mg/L) which contains the gene encoding catechol 1,2-dioxygenase (*CatAac*) from a bacterium *Acinetobacter calcoaceticus* ^40^, as shown in Fig. 3(B). The yields based on substrate consumption were 0.63 g ccMA/g vanillin and 0.48 g ccMA/g vanillin for the strains harboring construct 1 and with the expression of *CatApmt2* and *CatAac*, respectively. All the strains containing construct 1 yielded more than 50% higher titer of *cis, cis*-muconic acid than that of the strains harboring the construct 2. No PCA was detected in the fermentation broth of these strains. However, all the strains produced a detectable, but small amount of catechol (~2.9 mg/L) in the fermentation broth, as shown in Fig. 3(C).

Generally, the whole cell reaction mixture concentrates cell biomass and has higher cell density, which may produce the higher product titer and yield than regular fermentation. Therefore, in this study, we investigated the bioconversion of vanillin into *cis, cis*-muconic acid with the whole cell reaction mixture as well. The cell density in the whole cell reaction mixture was concentrated 20 times in the 1x M9 medium containing 10 g/L glucose and 0.5 g/L vanillin. As shown in Fig. 3(D), the concentrations of *cis, cis*-muconic acid achieved by whole cell bioconversion were ranged between 341 mg/L and 271 mg/L, which were about 63% and 136% higher than that obtained from strains containing construct 2 with catechol 1,2 dioxygenase CatAac and CatApmt2, respectively. For the strains expressing the construct 1 with *CatAac* or *CatApmt2* individually, the titers of *cis, cis*-muconic acid in the whole cell mixture were increased by 34% and 10%, respectively. The highest yield of ccMA based on the substrate consumption was 0.69 g ccMA/g vanillin. Compared to regular fermentation, no intermediate metabolites protocatechuate and catechol were detected in the mixture of whole cell, indicating the higher efficiency of whole cell bioconversion system than regular fermentation process.

### Bioconversion of syringate into gallate and pyrogallol

**Figure 4:**
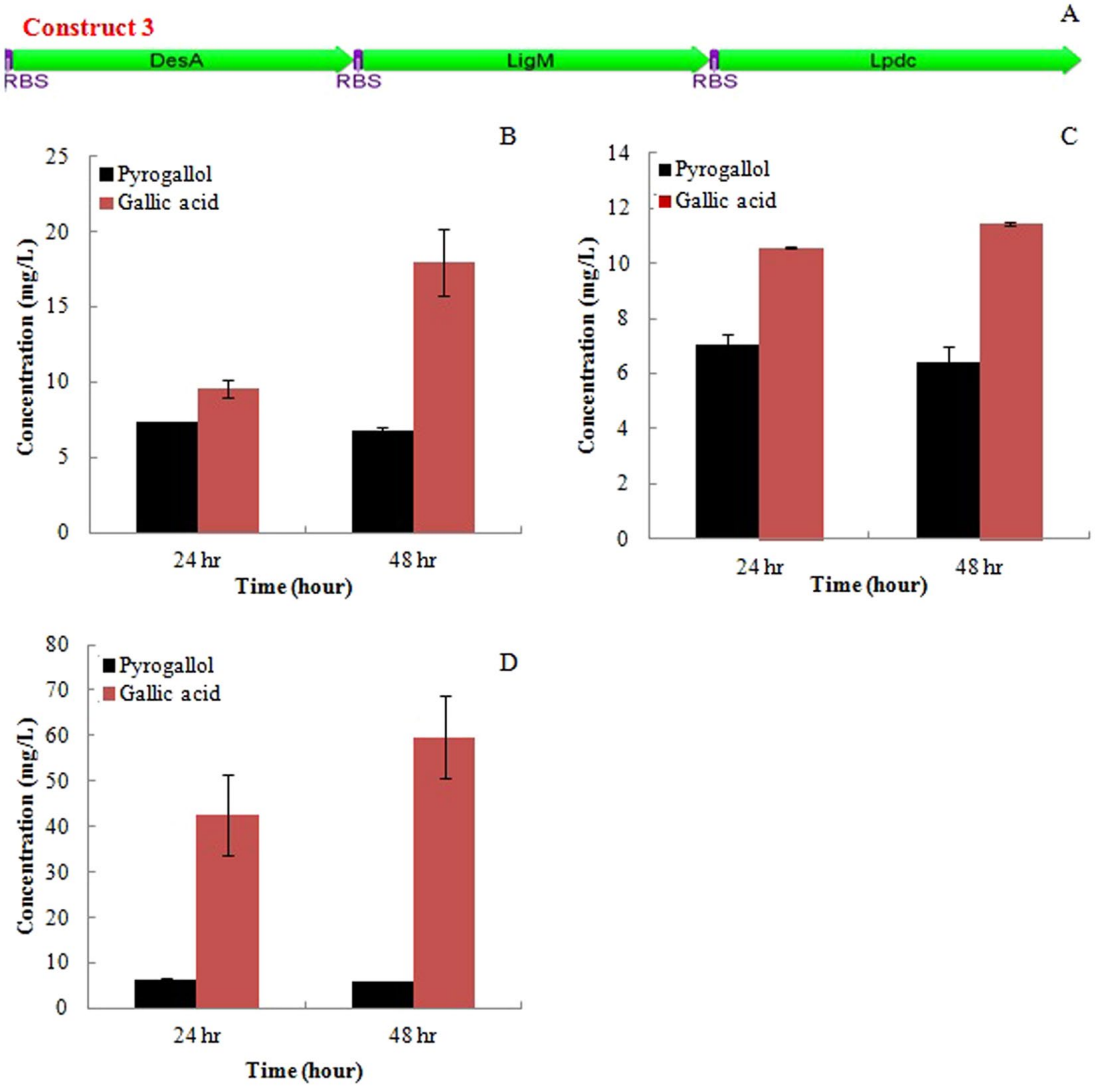
Bioconversion of syringate into pyrogallol. (**A**) Synthetic pathway for the bioconversion of syringate into pyrogallol; (**B**) pyrogallol and gallic acid concentration without the presence of tetrahydrofolate in the fermentation broth; (**C**) pyrogallol and gallic acid concentration with the addition of 100 µM tetrahydrofolate in the fermentation broth; (**D**) pyrogallol and gallic acid concentration in the whole cell bioconversion mixture.

In this study, we developed a novel pathway to bio-convert the syringate yielded from hydrogen peroxide catalyzed lignin into pyrogallol (Fig. 4). Two demethylase genes *desA* and *ligM* from *Sphingomonas paucimobilis* SYK-6 along with a decarboxylase gene *lpdc* from *Lactobacillus plantarum WCFS1* were co-expressed in the plasmid pBbE1a under the control of pTrc promoter, as shown in the construct 3 (as shown in Fig. 4). The results showed that the strain containing the construct 3 yielded small amounts of pyrogallol (~7.3 mg/L) as well as gallate (~18 mg/L) in the fermentation broth. No detectable amount of pyrogallol was observed in the fermentation broth of the wild-type strain under same cultural conditions. The yield of pyrogallol was significantly lower (7.3 mg pyrogallol/g syringate) in comparison to ccMA yield from vanillin. Both enzymes DesA and LigM are tetrahydrofolate dependent demethylase, indicating the need of tetrahydrofolate during syringate demethylation. However, the addition of cofactor tetrahydrofolate (100 μM) into fermentation broth didn’t improve the pyrogallol and gallic acid yields. The whole cell bioconversion of syringate generated similar yield pyrogallol (6.2 mg/L) as fermentation. However, the gallic acid titer was significantly higher than that produced in the fermentation broth, up to 59.6 mg/L gallate, corresponding to 59.6 mg gallate/g syringate.

### Bioconversion of extracted protocatechuate from plant biomass into *cis, cis*-muconic acid

**Figure 5:**
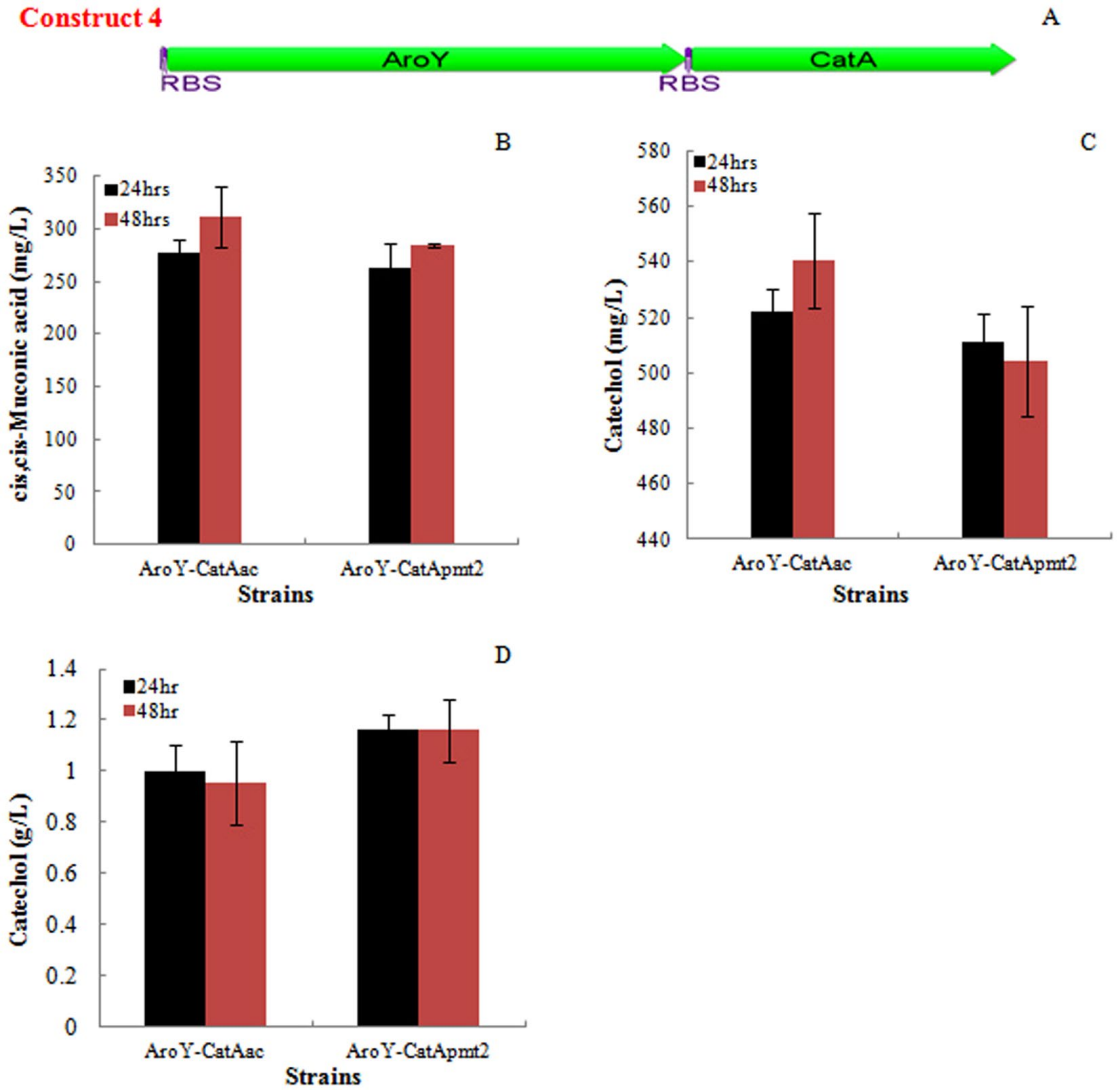
Bioconversion of extracted protocatechuate into *cis, cis*-muconic acid. (**A**) Synthetic pathway for the bioconversion of PCA into ccMA. Two pathway genes *AroY* and *CatA* were co-expressed under same promoter in the plasmid pBbE7K; (**B**) ccMA concentrations in the fermentation broth; (**C**) Catechol concentrations in the fermentation broth; (**D**) catechol concentration in the whole cell bioconversion mixture.

The metabolic biosynthesis of lignin building blocks depends on shikimate pathway that provides phenylalanine to the phenylpropanoid pathway^1, 3, 41, 42^. The genetic engineering of lignin biosynthesis to reduce the lignin content, modify the lignin content, and control the lignin deposition may decrease the plant cell wall recalcitrance and improve the efficiency of biomass pretreatment^2, 42, 43^. In this study, we succeeded in the accumulation of protocatechuate in the plant Tobacco through expressing a bacterial 3-dehydroshikimate dehydratase. With this achievement, we demonstrated a novel biochemical route of lignin valorization through the bioconversion of the methanol-water extracted PCA from engineered Tobacco into *cis, cis*-muconic acid with an engineered *E. coli* strain. To achieve this, the PCA was extracted and purified from the biomass powder of engineered Tobacco as described in the section of materials and methods. The highest yield of PCA achieved was at 1.45 mg/g plant stem. The extracted PCA was added into the fermentation broth of engineered *E. coli* culture and whole cell reaction buffer as a substrate at concentrations of 1 g/L and 2 g/L, respectively. As shown in the Fig. 5, various amounts of *cis, cis*-muconic acid were detected in the fermentation broth of strains expressing *aroY* encoding PCA decarboxylase and catechol 1, 2 dioxygenase genes *CatAac* or *CatApmt2*. Both strains yielded similar amount of *cis, cis*-muconic acid at the concentrations of 311 mg/L and 285 mg/L, in the fermentation broth. The highest yield of *cis, cis*-muconic was 0.311 g ccMA/g PCA, corresponding to 0.48 mg ccMA/g plant biomass. Besides the ccMA, the strains expressing *aroY* and *CatA* produced significant amount of catechol in the fermentation broth, up to 540 mg/L (0.54 g catechol/g PCA), compared to trace amount of catechol (~2.9 mg/L) accumulated in the culture of construct 1 and 2, indicating catechol 1, 2 dioxygenase is the limiting step in these two pathways. The whole cell reaction buffer didn’t yield obvious amount of muconic acid, as shown in Fig. 5(D). Instead, up to 1.16 g/L catechol was detected in the reaction buffer, representing 0.58 g catechol/g PCA, indicating the slow kinetics of catechol dioxygenase and possible severe inhibition of this enzyme by its substrate.

## Discussion

In this study, we have successfully demonstrated the first *de novo* production process for ccMA and pyrogallol in *E. coli* from lignocellulosic biomass through two hybrid biochemical routes. By combining chemically catalytic pretreatment of lignin, plant metabolic engineering, and the construction of heterologous synthetic pathways to convert vanillin and PCA into ccMA as well as syringate into pyrogallol, we achieved the yields of ccMA at 0.69 g ccMA/g vanillin and 7.3 mg pyrogallol/g syringate (route 1) as well as 0.31 g ccMA/g PCA (0.45 mg ccMA/g Tobacco stem, route 2), respectively.

Lignin is a complex and recalcitrant phenolic macromolecule with high structure heterogeneity that resists the microbial attack. One of the popular lignin depolymerization strategies is homogeneous acid/base-catalyzed deconstruction to carry out the fragmentation and separation of various lignin oligomers and monomers^11, 14, 44, 45^. Recently, the oxidative catalysis of lignin have resulted in the improved yields of oligomers, in particular, the vanillin yield was up to 10 wt% of lignin^11, 14, 19^. In the biochemical route 1, we applied the oxidative catalysis of the Kraft lignin with the addition of hydrogen peroxide as a catalyst for lignin depolymerization. As shown in Fig. 2, vanillin and syringic acid and their oxidized and reduced variants were produced as the main lignin oligomers. In nature, vanillin can be catabolized by some bacteria to yield the intermediates, such as protocatechuate and catechol, which further gets metabolized via beta-ketoadipate pathway to enters the TCA cycle^46^ and release CO_2_ as end product. However, many aromatic catabolizing microorganisms harbor the catechol 1,2-dioxygenase that opens the ring of catechol to yield ccMA^38–40^, which enables the possibility of ccMA bioproduction through engineering of genetically tractable microbial hosts with a heterologous pathway. Therefore, we introduced in *E. coli* the following two heterologous synthetic pathways: (1) By co-expression of the genes *ligV, ligM, aroY*, and *CatA*, to accomplish the bioconversion of vanillin into ccMA, and (2) By co-expression of *desA*, *ligM*, and *Lpdc* genes to achieve the bioconversion of syringate into pyrogallol.

The genes of *ligV* and *ligM* were chosen from the bacterium *Sphingomonas paucimobilis* SYK-6 since this strain has the ability to grow on various lignin-derived oligomers and monomers as the sole carbon source^46^. The vanillin dehydrogenase (LigV) conferred the ability of transforming vanillin into vanillate and was proved to be essential for normal growth of *S. paucimobilis* SYK-6 on vanillin^47^. Vanillate O-demethylase (LigM) converted the vanillate into PCA^48^, which can further be converted into catechol by PCA decarboxylase. The deletion of gene *ligM* retarded the growth of the strain, indicating that it is crucial for catabolism of vanillate as well^48^. The decarboxylase aroY showed the high enzyme activity on the PCA during the fermentation, the strain Bacillus subtilis WB800 expressing aroY yielded 0.68 g catechol/g PCA within 20 hours’ culture (data not shown). Both tested CatA isomers showed activity on catechol in the synthetic pathway, although the strain expressing catechol-1, 2-dioxygenase from *Pseudomonas putida mt-2* produced 31% higher yield of ccMA from vanillin in the fermentation than the catechol-1, 2 dioxygenase from *Acinetobacter calcoaceticus*. In spite of the fact that the PCA decarboxylation was considered as a rate-limiting step in ccMA biosynthesis from sugars^26–28^, the overexpression of aroY in the pathway not only didn’t yield the higher concentration of ccMA but reduced the titer of ccMA in the fermentation for both CatAac and CatApmt-2. Interestingly, a various amounts of catechol were detected in the fermentation broth from both biochemical route 1 and 2, indicating the catechol-1, 2-dioxygenase is the limiting rate step in our case, particularly for biochemical route 2, in which 100% higher concentrations of catechol were detected in the fermentation broth than ccMA.

The gene *desA* encoding syringate O-demethylase was chosen from the soil bacterium *Sphingomonas paucimobilis* SYK-6 as well, catalyzing the demethylation of syringate into 3-O-methylgallate (3MGA) that is further converted into gallate by another demethylase LigM^49, 50^. In the syringate catabolic pathway of the bacterium *S. paucimobilis* SYK-6, the resulting gallate is degraded by a dioxygenase to finally enter the TCA cycle^49, 50^. A gallate decarboxylase (*Lpdc*) gene^51^ was introduced in *E. coli* to drive the decarboxylation of gallate into the pyrogallol as a value-added compound with the potential treating the cancer. The strains containing the introduced pathway produced detectable amounts of pyrogallol (7.3 mg/L) and gallic acid (18 mg/L) at room temperature although the yield of pyrogallol was relatively low. Both enzymes DesA and LigM are tetrahydrofolate-dependent *O*-demethylase. Eiji Masai *et al*. had demonstrated that both DesA and LigM enzymes were only active at the presence of tetrahydrofolate to convert syringate into 3-*O*-methylgallate^52^. However, we didn’t observe the significant difference between pyrogallol yields with/without addition of tetrahydrofolate into fermentation broth. Most likely, either the rich medium LB contains enough tetrahydrofolate, the *E. coli* can synthesize enough amount of tetrahydrofolate, or its uptake becomes limiting. A lactic acid bacterium *Lactobacillus plantarum* has three non-oxidative aromatic acid decarboxylases genes (*LpdB*, *LpdC*, and *LpdD*) in the chromosome and the enzyme Lpdc was confirmed as the only protein required to yield gallate decarboxylase activity^51^. However, the recombinant LpdC presented low gallate decarboxylase activity even through the enzyme LpdC was produced in high yield^51^. Most likely, it is the reason why the pyrogallol yield was relatively low. The gallic acid was accumulated at a high titer in the whole cell reaction, confirming the gallate decarboxylase activity is the limiting rate step in this engineered pathway.

Lignin, a complex aromatic biopolymer, is a major component of the plant cell wall, conferring structural recalcitrance and prevents the release of sugars as the renewable carbon source for the production of bioproducts. Therefore, there are extensive studies on the genetic manipulation of lignin biosynthesis pathway to reduce lignin content, to control lignin deposition, to relieve the structural recalcitrance and to improve biomass saccharification efficiency^1, 2, 41, 42, 53^. In the hybrid biochemical route 2, the shikimate biosynthesis pathway of plant Tobacco was engineered to convert 3-dehydroshikimate, an intermediate of the shikimate pathway, into PCA for subsequent bioconversion into ccMA. The methanol-water solvent extraction of PCA from the engineered plant Tobacco yielded 1.45 mg PCA/g mature stem. The genes encoding aroY and CatA were co-expressed to convert PCA into ccMA, which yielded 0.45 mg ccMA/g plant tissue with 0.78 mg catechol/g plant tissue. The existence of large amounts of catechol in the fermentation broth indicated that open-ring reaction of catechol catalyzed by CatA is the limiting step for the ccMA bioproduction. A recent study showed that protein engineering of the CatA from *Acinetobacter Sp. ADP1* improved its activity by 10-times in comparison to the wild-type one^38^. With the application of the results from this study, the ccMA yield based on biochemical route 2 can be improved further.

With the aim of improving the biorefinery economics, we have established two hybrid biochemical routes to convert the polymeric lignin into (1) ccMA, a precursor for the building blocks for various commodity plastics, and (2) pyrogallol, a potential drug precursor for treating cancer. For the first time, we have demonstrated that the pyrogallol can be biosynthesized from lignin derived aromatics with a heterologously introduced pathway in *E. coli*. Our approach focused on vanillate and syringate; two major products observed in most of the catalytic depolymerization of lignin that are also the starting substrates for SYK-6 central metabolic pathways. The biochemical route 1 combined oxidatively catalyzing the Kraft lignin to produce vanillin and syringate with the heterologous pathway reconstruction in *E. coli* to convert those vanillin and syringate into ccMA and pyrogallol, respectively. In the Vardon *et al*. study, an aromatics assimilation strain *Pseudomonas putida* KT2440 was engineered through the introduction of genes *aroY* and *CatA* to convert the PCA to *cis, cis*-muconic acid and the deletion of genes *PcaHG* and *CatBC* for PCA decarboxylation and catechol ring opening, respectively. With the integration of intrinsic aromatics degradation pathway and introduction of foreign genes, the engineered strain funneled multiple aromatics, including coniferyl alcohol, benzoate as well as phenol in the lignocellulosic liquor to *cis, cis*-muconic acid as precursor for the following adipic acid production^28^. Similarly, in Linger *et al*. study, the pretreated lignocellulosic liquor containing lignin-derived aromatics was converted to biopolymer by the engineered aromatics assimilation strain *Pseudomonas putida* KT2440^8^. Due to the high heterogeneity and the complex structure of the lignin, the current technologies of lignin valorization can only bioconvert a small portion of lignin in the pretreated biomass liquor to value-added products. As of yet, there has been no demonstration of biotransformation of pretreated lignin liquor directly. Compared to the work of Vardon *et al*. and Linger *et al*., in this study, a muconic acid biosynthesis pathway was heterologously reconstructed into the well-understood model organism *E. coli*. The polymeric lignin was depolymerized and the aromatic compound vanillin was extracted as a model substrate for the engineered chassis, achieving the muconic acid production from polymeric lignin under the developed hybrid Chem-Synbio route directly. Compared to previous studies^8, 28^, the purification of individual aromatic compound from chemical depolymerization of lignin was introduced before the bioconversion of them into value-added compounds. One of distinct benefits of this study is that the final purification of target compound could be much easier since culture broth will not contain the complex lignin-derived aromatics yielded from the pretreatment of lignocellulosic biomass, which was used as the substrate in the previous studies^8, 28^, although the major portion of them may be consumed during the fermentation. This study offers the research community a first study of developing a novel Chem-Synbio route for lignin valorization that combined a more efficient thermochemical depolymerization of lignin and high selective production of target compound from aromatics purified from pretreated lignin liquor. Heterogeneity of the lignin is a big challenge for making large volume of valuable chemicals from lignin. To overcome this issue, the biochemical route 2 combined the lignin bioengineering to produce the intermediate compound PCA along with strain engineering to convert PCA into ccMA. This biochemical route eliminates the depolymerization and separation/extraction challenges as well as heterogeneity problem. Compared to aromatics tolerant strain *Pseudomonas putida* KT2440 in the previous studies^8, 28^, the strain *E. coli* used in this study is less tolerant to the aromatic compounds. However, *E. coli* has well-established genetic tools, which can be intensively engineered to improve strain performance. For example, the genetic engineering tool will allow successful engineering of the *E. coli* with higher aromatics tolerance, higher aromatics uptake rate, and higher target compound productivity. In addition, the higher growth rate of *E. coli* compared to *P. putida* makes it a suitable chassis for the large-scale production. There are other microbes such as *Bacillus ligninifilus* L1^54^ and *Sphingomonas paucimobilis* SYK-6^16^ that show the ability to assimilate aromatics as well as the tolerance to aromatics and may prove to be valuable microbial hosts for lignin valorization, In this study using *E. coli*, for the unoptimized processes, the route 1 yielded 0.69 g ccMA/g vanillin and 7.3 mg pyrogallol/g syringate while the route 2 produced 0.31 g ccMA / g PCA (0.45 mg ccMA /g Tobacco stem) and the catechol yield at 0.79 mg catechol/g plant tissue. Although the yield of ccMA and pyrogallol were less impressive in this unoptimized approach, hereby, we demonstrated the concept and the feasibility of bioproduction of high-value ccMA and pyrogallol as value-added chemicals and potential pharmaceuticals from lignin in *E. coli*, thereby serving as a promising route for lignin valorization.

## Methods

### Catalytic depolymerization of Kraft lignin

In a typical procedure, 1 g of Kraft lignin (Sigma, MO) was taken in a glass pressure tube and was solubilized in 4.5 mL of 40% NaOH at room temperature. To homogenize the solution of lignin, 4.5 mL of hydrogen peroxide (30% in water) was added dropwise at room temperature to final volume 10 mL of lignin solution (10 wt%). After the completion of addition, the reaction mixture was stirred at 80 °C for 4 h. Then the reaction mixture was cooled and the pH was adjusted to 7 using 6 N HCl. The reaction mixture was filtered and the filtrate was used as the depolymerized lignin source.

### Tobacco transformation

A DNA fragment containing the NOS promoter (*pNOS*) followed by the gene coding for the DsRed2 fluorescent protein and the NOS terminator (*tNOS*) was synthesized (Genescript, Piscataway, NJ), amplified by PCR using the DsRed-F and DsRED-R primers (Table 2), and inserted by In-Fusion cloning (Clontech Laboratories, Inc., Mountain View, CA) into the pTKan-*pC4H::schl-qsuB* vector^42^ previously digested with *Sma*I to generate the pTKan-*pC4H::schl-qsuB-DsRed2* construct. Tobacco (*Nicotiana tabacum* L) was transformed with *Agrobacterium tumefaciens* (strain *CV3101*) harboring the pTKan-*pC4H::schl-qsuB-DsRed2* construct using the leaf disc method^55^ and as previously described^56, 57^. All Tobacco *in vitro* cultures were maintained in a growth chamber at 26 °C with 16/8 h light photoperiod at 40 µE m^−2^ s^−1^. Transgenic plantlets expressing DsRED2 were transferred to soil and grown in a chamber under the same conditions.

**Table 2:** Primers used in this study.

pBbE7k-AroY-F	5-TCAGAATTC *ACCATTTCATTTAAGGACTACCACCGCAAC*ATGACAGCCCCTATTCAAGAC -3
pBbE7k-AroY-R	5- GGATCC AGA TCT TTA TTT AGC GGA GCC TTG ATT TTT T-3
pBbE7k-CatAac-F	5-ATTTCAAGATCT *TCGTCCCATAAATTCAAAGGAGACTACC*ATGAACAGACAGCAAATCGACG-3
pBbE7k-CatAac-R	5-AGATCTGGATCC TTA GGC TGA GGC TCT TCT TCT-3
pBbE7k-CatApmt2-F	5-GAATTCAGATCT *TTAACCAACCGATACACGTATAAGAATTTAGTAC*ATGACAGTGAAAATCAGC CATACAGC-3
pBbE7k-CatApmt2-R	5-CTCGAGGGATCC TTA TCC TTC TTG TAA TGC TCT CGG G-3
pBbE1a-ColE1-Amp_G-F1	5-GGA TCC AAA CTC GAG TAA GGA TCT CCA GGC-3
pBbE1a-ColE1-Amp_G-R1	5- CAT TCG ATG GTG TCG ACG TCA GGT GGC ACT-3
pBbE1a-LacI-ptrc-LacO-G-F2	5-AGT GCC ACC TGA CGT CGA CAC CAT CGA ATG-3
pBbE1a-LacI-ptrc-LacO-G-R2	5-GAA TTC TGA AAT TGT TAT CCG CTC ACA ATT CCA CAC-3
pBbE1a-LigV-Gib-F	5- GTG TGG AAT TGT GAG CGG ATA ACA ATT TCAGAATTC CCA TAG CCC AAC ATA GAA TAA GGT AC-3
pBbE1a-LigV-Gib-R	5-CTC CTT GCT TTA ATG GTG GGA AAG AGATCT TTA AAT CGG AAA ATG GCCCGG TTG G-3
pBbE1a-LigM-Gib-F	5-GAATTC AGATCTCTTTCCCACCATTAAAGCAAGGAGTAAATAATTA ATG AGC GCT CCG ACC AAC CTG-3
pBbE1a-LigM-Gib-R	5-CGG TGG TAG TCC TTA AAT GAA ATG GT GGATCC TTA CGC GGT CAC CGC CGC TTT A-3
pBbE1a-AroYA-Gib-F	5-CTCGAG GGATCC ACCATTTCATTTAAGGACTACCACCGCAAC ATG ACA GCC CCT ATT CAA GAC -3
pBbE1a-AroYA-Gib-R	5- GTT TTA TTT GAT GCC TGG AGA TCC TTA CTCGAG TTA TTT AGC GGA GCC TTG ATT TTT T-3
pBbE1a-DesA-GibF	5-GTG TGG AAT TGT GAG CGG ATA ACA ATT TCAGAATTC AAGTTTACCCCCTAATTTCAAAGTCGGTTCTTCT ATG GCA AAA TCT CTT CAG GAC G-3
pBbE1a-DesA-GibR	5-CTC CTT GCT TTA ATG GTG GGA AAG AGATCT TTA GGC TTT TTT CGT CCG CCA G-3
pBbE1a-LigM-GibF	5-TAA AGATCTC TTTCCCACCATTAAAGCAAGGAGTAAATAATTA ATG AGC GCT CCG ACC AAC CTG-3
pBbE1a-LigM-GibR	5- CTT cgt tct act tac ttc ccc ttg ata gat agg gc GGATCC TTA CGC GGT CAC CGC CGC TTT AC-3
pBbE1a-LpdC-GibF	5-GGATCC GCCCTATCTATCAAGGGGAAGTAAGTAGAACGAAG ATG GCG GAA CAG CCT TGG GAC C-3
pBbE1a-Lpdc-GibR	5-GAT GCC TGG AGA TCC TTA CTC GAG CTCGAG TTA TTT CAG GTA TTT TTC CCA ATC CGC AAC-3
DsRed-F	5-tcgagctcggtacccggGATACATGAGAATTAAGGGAGTC-3
DsRed-R	5′-agaagcttggtacccggGAGCTTGCATGCCGGTCGATC-3

### Protocatechuate extraction from the biomass of the engineered Tobacco

PCA was extracted from engineered Tobacco following the procedure described by Eudes *et al*.^42^. Briefly, 1 g of powder was mixed with 2 mL of 80% (v/v) methanol for 15 mins at 1400 rpm, 70 °C. This step was repeated four times. The accumulated extracts were cleared by centrifugation at 20000 g, 5 minutes, 20 °C. The supernatant was mixed with 4 mL of HPLC grade water and filtered through Amicon ultra centrifugal filters (10000 Da MW cut off, EMD Millipore, Billerica, MA, USA). Filtered extracts were analyzed by HPLC for PCA measurements.

### Plasmids construction

The genes (*ligV*, *ligM*, *desA*, *aroY*, *CatAac*, *CatApmt2, and Lpdc*) in this study were listed in the Table 1. All the genes were codon optimized for *E. coli* expression and synthesized by Genscript. The ribosome binding site for each gene was calculated and optimized using the RBS calculator developed by Salis Lab at Penn State University. The gene *aroY* was cloned into the plasmid pBbE7k under the restriction cutting site *Eco*RI/*Bgl*II to achieve the plasmid pBbE7k-aroY. The genes *CatAac* and *CatApmt2* were amplified and subcloned into plasmids pBbE7k, pBbE7k-aroY under and *Bgl*II */Bam*HI to achieve the plasmids pBbE7k-CatA and pBbE7k-aroY-CatA, respectively. The genes *ligV*, *LigM*, and *aroY* were amplified and assembled into plasmid pBbE1a by Gibson assembly to obtain plasmid pBbE1a-VMY. The genes *desA*, *ligM*, and *Lpdc* were assembled into plasmid pBbE1a by Gibson assembly as well to achieve plasmid pBbE1a-AML. All the primers used for gene amplifications were listed in the Table 2.

All the synthesized DNA sequences (*LigV*, *LigM*, *DesA*, *AroY*, *CatA-pmt2* and *CatA-ac*) were submitted into GenBank with the accessions: KX774254, KX774255, KX774256, KX774257, KX774258, KX774261, KX774262, respectively.

### Strain, medium and cultivation conditions

The strain DH1 was obtained from Joint Bioenergy Institute by the courtesy of Dr. Taek Soon Lee. The plasmids pBbE1a-VMY and pBbE7k-aroY-CatA or pBbE7k-CatA were co-transformed into strain DH1 for the bioconversion of vanillin to *cis*, *cis*-muconic acid. The plasmids pBbE7k-aroY-CatA were transformed into strain DH1 to achieve the bioconversion of the protocatechuate extracted from engineered biomass to *cis*, *cis*-muconic acid as well. The plasmid pBbE1a-AML was transformed into strain DH1 for the bioconversion of syringate into pyrogallol. The positive transformants of strain DH1 were cultivated in 5 mL of LB medium containing corresponding antibiotics (100 μg/mL ampicillin, 25 μg/mL kanamycin), overnight. One mL of overnight culture were transferred into 20 mL of fresh LB medium containing 20 g/L glucose with the same antibiotics and cultured at 220 rpm, 37 °C until the OD reached 0.8. Then, the cultures were induced by the addition of IPTG at 1 mM for another 18 hours. The vanillin, protocatechuate, syringate were added into culture at the concentration of 0.5 g/L or 1 g/L, respectively, for the muconic acid and pyrogallol production. The strain DH1 without plasmids was cultured under same conditions as the negative control except that antibiotics were omitted. The samples of the cultures were taken at different time intervals for the further analysis. All the experiments were performed in duplicates. All the strains were listed in the Table 3.

**Table 3:** Strains utilized in this study.

Strains	Plasmids contained	Pathway genes	construct
VMY-YCatAac	pBbE1a-VMY, pBbE7k-aroY-CatAac	*LigV*, *LigM*, *AroY*, *CatAac*	2
VMY-CatAac	pBbE1a-VMY, pBbE7k-CatAac	*LigV*, *LigM*, *AroY*, *CatAac*	1
VMY-YCatApmt2	pBbE1a-VMY, pBbE7k-aroY-CatApmt2	*LigV*, *LigM*, *AroY*, *CatApmt2*	2
VMY-CatApmt2	pBbE1a-VMY, pBbE7k-CatApmt2	*LigV*, *LigM*, *AroY*, *CatApmt2*	1
AroY-CatAac	pBbE7k-AroY-CatAac	*AroY*, *CatAac*	4
AroY-CatApmt2	pBbE7k-AroY-CatApmt2	*AroY*, *CatApmt2*	4
AML	pBbE1a-AML	*desA, LigM, LpdC*	3
CV3101	pTKan-*pC4H::schl-qsuB-DsRed2*	*QsuB*	in this study

### Whole cell bioconversion of vanillin, PCA into *cis, cis*-muconic acid as well as syringate into pyrogallol

The single colony of strains DH1 expressing corresponding construct (construct 1, 2, 3, or 4) were cultured in 5 mL of LB medium containing corresponding antibiotics (100 μg/mL ampicillin, 25 μg/mL kanamycin), overnight. Two mL of overnight culture were transferred into 200 mL of fresh LB medium containing 20 g/L glucose with the corresponding antibiotics and cultured at 220 rpm, 37 °C until the OD reached 0.8. Then, the cultures were induced by the addition of IPTG at 1 mM for another 18 hours at 30 °C. Then, the culture was spun down, the supernatant decanted, and the cells re-suspended in 4 mL of 1X M9 medium^58^ containing 10 g/L glucose. 1 mL of vanillin, PCA, or syringic acid stock was added into the reaction mixture at the final concentration of 0.5 g/L vanillin, 2 g/L PCA, 1 g/L syringic acid, respectively. The whole cell mixtures were incubated at 30 °C, 180 rpm. The samples were taken every 24 hours for HPLC analysis.

### Analytical Methods

Identification of chemical compounds in depolymerization products was carried out using an Agilent 6890 N gas chromatography equipped with Agilent 5973 N mass spectrometry. The capillary column used was an Agilent DB-5MS (30 m × 0.25 mm × 0.25 μm). Injection temperature was 250 °C and oven temperature was programmed to hold at 50 °C for 1 min, ramp to 300 °C at 10 °C/min and then hold for additional 1 min.

In terms of fermentation broth, the samples were centrifuged at 14000 rpm, 4 °C for 15 mins and the supernatants were filtrated through 0.2 μm PTFE membrane before analysis. The concentrations of vanillin, protocatechuate, catechol, muconic acid, syringic acid, gallic acid, and pyrogallol in the samples were analyzed by a high-pressure liquid chromatography (HPLC, Agilent 1100) using a Rezex ROA column (Phenomenex, San Jose, CA) at 65 °C under UV detector (200 nm, 220 nm) for 65 mins. The mobile phase was 0.005 N sulfuric acid at the flow rate of 0.5 mL/min.
